# Mutation spectrums of *TSC1* and *TSC2* in Chinese women with lymphangioleiomyomatosis (LAM)

**DOI:** 10.1371/journal.pone.0226400

**Published:** 2019-12-19

**Authors:** Jie Liu, Weiwei Zhao, Xiaohua Ou, Zhen Zhao, Changming Hu, Mingming Sun, Feifei Liu, Junhao Deng, Weili Gu, Jiaying An, Qingling Zhang, Xiaoxian Zhang, Jiaxing Xie, Shiyue Li, Rongchang Chen, Shihui Yu, Nanshan Zhong

**Affiliations:** 1 Department of Pulmonary and Critical Care Medicine, The First Affiliated Hospital of Guangzhou Medical University, Guangzhou, Guangdong, China; 2 Guangzhou Institute for Respiratory Health, Guangzhou, Guangdong, China; 3 State Key Laboratory of Respiratory Diseases, Guangzhou, Guangdong, China; 4 National Clinical Research Center for Respiratory Disease, Guangzhou, Guangdong, China; 5 Guangzhou KingMed Diagnostics Group Co., Ltd, Guangzhou, Guangdong, China; 6 Clinical Genome Center, KingMed Center for Clinical Laboratory Co., Ltd, Guangzhou, Guangdong, China; 7 KingMed College of Laboratory Medicine, Guangzhou Medical University, Guangzhou, Guangdong, China; 8 Guangzhou KingMed Translational Medicine Institute Co., Ltd, Guangzhou, Guangdong, China; 9 Department of Pulmonary and Critical Care Medicine, Shenzhen People's Hospital, Shenzhen, Guangdong, China; 10 KingMed JianShi Innovation Institute (Guangzhou) Co., Ltd, Guangzhou, Guangdong, China; German Cancer Research Center (DKFZ), GERMANY

## Abstract

The aim of our study was to elucidate the landscapes of genetic alterations of *TSC1* and *TSC2* as well as other possible non-*TSC1/2* in Lymphangioleiomyomatosis (LAM) patients. Sixty-one Chinese LAM patients’ clinical information was collected. Tumor biopsies and matched leukocytes from these patients were retrospectively analyzed by next generation sequencing (NGS), chromosomal microarray analysis (CMA), and multiplex ligation-dependent probe amplification (MLPA). Eighty-six *TSC1/2* variants were identified in 46 of the 61 LAM patients (75.4%) in which *TSC2* and *TSC1* variants were 88.37% and 11.63% respectively. The 86 variants are composed of (i) 52 single nucleotide variants (SNVs) (including 30 novel variants), (ii) 23 indels (including 21deletions, and 2 insertions), (iii) a germline duplication of exon 31–42 of *TSC2*, (iv) a 2.68 Mb somatic duplication containing *TSC2*, and (v) 9 regions with copy-neutral loss of heterogeneity (CN-LOHs) present only in the LAM patients with single *TSC1/2* mutations. Sixty-one non-*TSC1/2* variants in 31 genes were identified in 37 LAM patients. Combined applications of different techniques are necessary to achieve maximal detection rate of *TSC1/2* variants in LAM patients. Thirty novel *TSC1/2* variants expands the spectrum of *TSC1/2* in LAM patients. Identification of 61 non-*TSC1/2* variants suggests that alternative genes might have contributed to the initiation and progression of LAM.

## Introduction

Lymphangioleiomyomatosis (LAM) is a rare, low-grade, destructive, and metastasizing neoplasm that mainly affects women, usually during their childbearing years, and occasionally affects men [1–3]. Clinical features are characterized by progressive cystic destruction of the lung parenchyma, recurrent pneumothorax, development of fluid-filled lymphatic cystic structures (lymphangioleiomyomas), chylothorax, and abdominal angiomyolipomas. Clinical diagnosis can be routinely achieved based on symptoms and results from radiological scanning, pathological biopsies and subsequent immuno-histochemical staining [1,4–7].

LAM is known to be caused by mutations in the tuberous sclerosis complex (TSC) genes, *TSC1* located on chromosome 9q34 encoding hamartin or *TSC2* located on chromosome 16p13 encoding tuberin [8–10]. Mutations in both hamartin and tuberin lead to inactivation of the tuberin-hamartin complex, resulting in increased activity of a kinase known as the mammalian target of rapamycin (mTOR) leading to abnormal proliferation of smooth muscle-like cells (LAM cells), the underlying causes of a variety of pathological findings.

LAM can occur sporadically (S-LAM) or in association with TSC (TSC-LAM), an autosomal dominant syndrome characterized by multisystem growth of hamartoma (such as, lung, brain, kidney, heart, retina and skin) as well as neurological features (such as, seizures, autism, and intellectual disabilities). Consistent with the Knudson’s “two-hit” theory for explaining the tumorigenesis in which usually through a mutation in one allele coupled with loss of the remaining wild-type allele [11,12], S-LAM develops arising from two somatic mutations in *TSC1/2* while TSC-LAM have one germline mutation and one acquired mutation [13,14]. However, two hits (mutations) in *TSC1*/*2* are not always observed in patients with definite diagnosis of LAM, and the underlying reasons haven’t been comprehensively investigated yet.

In this study, we systematically investigated 61 Chinese female LAM patients focusing on unveiling the landscapes of genetic alterations of *TSC1*/*2* as well non-*TSC1*/*2* in their genomes. A comprehensive landscape of *TSC1/2* genetic alterations leading to the tumorigenesis of LAM were identified, including single-nucleotide variants (SNVs), small insertions or deletions (indels), copy number variants (CNVs), and regions of copy-neutral loss-of-heterozygosity (CN-LOH), with more than one-third of the variants being novel mutations. Meanwhile, we identified a long list of variants in non-*TSC1*/*2* genes, and proposed additional mechanisms participating in the initiation and progression of S-LAM.

## Materials and methods

### Patients

Seventy-six Chinese patients with definite diagnosis of LAM disease were initially enrolled and 61 of them were retrospectively analyzed in this project. All of these patients were diagnosed, treated, and followed up in the Guangzhou Institute of Respiratory Health from April of 2011 to Dec. of 2017 according to the clinical guidelines [2,15]. Patients’ general information, disease history, family history, radiological findings, pathological and relevant laboratory testing results were collected from their medical records and summarized in S1 and S2 Tables. For this retrospective study, the Medical Ethics Committee of the First Affiliated Hospital of Guangzhou Medical University waived the requirement to obtain consent from patients included in the research project on April 22, 2019,with approval number:2019-K-18, considering the fact that all data were retrospectively analyzed anonymously.

### Specimens and DNA extractions

**Fig 1:**
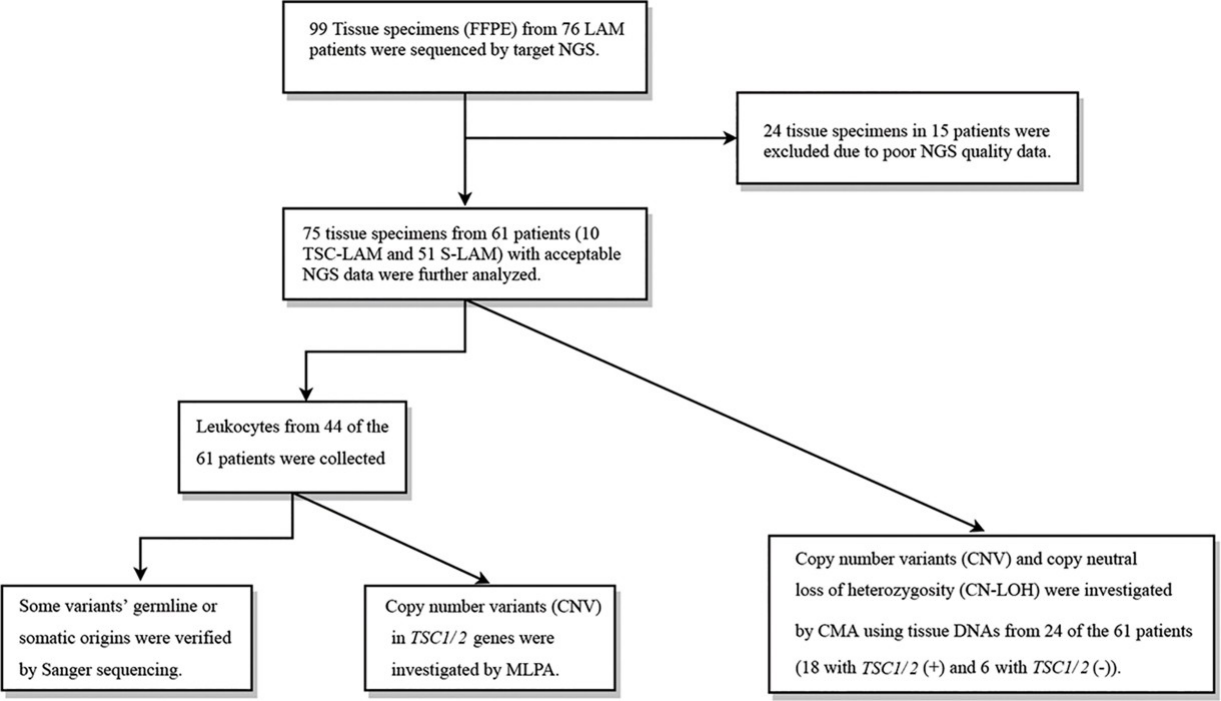
Outline of the DNA samples tested in the LAM patients.

Ninety-nine formalin-fixed paraffin-embedded (FFPE) specimens were collected from the 76 LAM patients. These FFPE specimens were prepared as part of routine clinical care from different biopsies with the majority of them from lung and the some from kidney, retroperitoneal, pelvic cavity, and uterus (Fig 1, Table 1).

**Table 1:** Tissue biopsy specimens obtained from the 61 LAM patients.

Specimens (number of patients)	Sampling spots	Number of patients	Number of specimens
1 specimen (49)	lung	40	40
	retroperitoneal	1	1
	pelvic cavity	1	1
	kidney	7	7
2 specimens (11)	2 specimens from lungs	7	14
	1 from lung and 1 from kidney	1	2
	1 from lung and 1 from uterus	1	2
	2 specimens from kidney	2	4
4 specimens (1)	3 from lung and 1 from retroperitoneum	1	4
Total		61	75

Before DNA extraction, biopsy specimens were reviewed by at least one certified clinical pathologist to assess the presence of LAM cells. DNAs from these biopsy specimens were isolated using GeneRead DNA FFPE kit (QIAGEN, Hilden, GER). After evaluation of the DNA qualities, 75 of the 99 biopsy specimens from 61 of the 76 LAM patients were qualified for further NGS analysis, leaving 24 specimens from 15 LAM patients being excluded from this study. The 61 patients with 75 biopsy specimens are composed of 49 patients with a single specimen (40 patients with single lung specimens, 7 patients with single kidney specimens, one patient with a single retroperitoneal specimen, and one patient with a single pelvic cavity specimen), 11 patients with two separate specimens (7 patients with 2 lung specimens, 2 patients with 2 kidney specimens, 1 patient with 1 lung specimen plus a kidney specimen, and 1 patient with 1 lung specimen plus a uterus specimen), and 1 patient with 4 specimens (3 lung specimens plus a retroperitoneal specimen). We could also extract leukocyte DNAs from 44 of the 61 LAM patients using QIAamp DNA Mini Kit (QIAGEN, Hilden, GER) (Fig 1).

### Target capture sequencing

A custom gene panel (S3 Table) were designed for sequencing the genomic DNA (gDNA) extracted from the biopsies. The gene panel encompasses 76 key genes including *TSC1* and *TSC2* related to tumorigenesis in a variety of malignant tumors. gDNA library preparations were performed with KAPA Hyper Plus Kit, (Kapa Biosystems, Wilmington, USA). The library mixtures were performed by a hybrid capture-based procedure using xGen Lockdown Reagent (Integrated DNA Technologies, Coralville, USA). The captured libraries were sequenced on an Illumina Miseq or Nextseq500 sequencer (Illumina, San Diego, USA) for 150 cycles of paired-end reads with mean target coverage >1000x and minimum depth of coverage ≥200x. Following sequencing, fastq files were generated with Illumina software, sequencing reads were aligned to the GRCh37/hg19 assembly with BWA-MEM, and variants were filtered, called, and annotated using GATK, Pisces and ANNOVAR, respectively. Variant allelic fractions (VAF) of somatic variants (SNVs or indels) down to 1.0% were achieved, and a total read count ≥200 and a variant read count ≥5 were set up as the minimum numbers required in this study. The clinical significance of the germline and somatic variants were assessed according to the published guidelines and related database LOVD2.0 [16–20]. Data have been deposited at the European Genome-phenome Archive, which is hosted by the European Bioinformatics Institute, under study accession number EGAS00001003534.

### Sanger sequencing

Sanger sequencing for DNAs extracted from leukocytes was used to characterize some genetic variants identified by next generation sequencing (NGS) from tumor DNAs in which it’s difficult to determine whether these variants were germline-originated or somatically-mutated in *TSC1*/2. Based on our validated pipelines, a variant with VAF ≤ 30% is considered somatic while a variant with 30% ≤ VAF ≤ 70% is considered germline for both variants identified in *TSC1/2* and non-*TSC1/2*. To further validate the origins of the variants in *TSC1/2*, Sanger sequencing was applied using DNAs extracted from leukocytes in 21 patients with 26 *TSC1/2* variants identified in their tumor DNAs by NGS with VAF ranging from 10% to 75%. Sanger sequencing were performed on ABI-3730xl system (Thermo Fisher Scientific, Carlsbad, USA).

### Chromosomal microarray analysis (CMA)

Biopsied DNA samples from 24 LAM patients were performed by CMA method to detect CNVs and CN-LOH according to the manufacturer’s instruction (Thermo Fisher Scientific, Carlsbad, USA). The CMA platform applied is composed of 550,000 non-polymorphic CNV probes and more than 200,000 Single nucleotide polymorphism (SNP) probes with an average resolution of 100 kb. This SNP array platform can detect both CNVs and CN-LOH. All data were visualized and analyzed with the Chromosome Analysis Suite (ChAS) software. The analysis was based on the reference genome of GRCh37/hg19 assembly. The clinical significance of CNVs and CN-LOHs were assessed according to the published guidelines [21,22].

### Multiplex Ligation-dependent probe amplification (MLPA)

Leukocyte DNAs extracted from the 44 LAM patients were tested by MLPA to identify CNVs of *TSC1/2* with SALSA MLPA probe-mix P124-C1 (*TSC1*) and P046-C1 (*TSC2*) according to the manufacturer’s instruction (MRC-Holland; Amsterdam, The Netherlands).

### Statistical methods

Several statistical methods were used for analyzing the data in this study. Fisher's exact test was performed for comparing the status of CN-LOHs in LAM patients carrying different numbers of *TSC1/2* variants. Chi-square testing was used for comparing the clinical information and genetic results of *TSC1/2* in the TSC-LAM and S-LAM. One-way ANOVA test was used for comparing novel and previously identified in *TSC1/2* variants. All tests were two-tailed, with statistical significance defined as *P<0*.*05*. These statistical analyses were performed with Graphpad Prism 6.0 software and IBM SPSS Statistics 22.

## Results

### Clinical, imaging, pathological, and biochemical findings

Of the 61 patients, 10 (16.4%) of them were diagnosed as TSC-LAM according to the recommendations from the International Tuberous Sclerosis Complex Consensus Group [23,24], and 5 (8.2%) of them had a family history of TSC. Concurrent presence of lung adenocarcinoma previously diagnosed in a TSC-LAM patient (ID052) was validated (S1 Fig).

The most common clinical manifestations in the 61 LAM patients include breathlessness in 41 (67.2%) patients (9 TSC-LAM, 32 S-LAM), spontaneous pneumothorax in 34 (55.7%) (5 TSC-LAM, 29 S-LAM), and chylothorax in 3 (4.9%) patients (0 TSC-LAM, 3 S-LAM). Chest Computer Tomography (CT) scans identified diffuse cystic features and multiple bullies in all the 61 patients including 6 cases (9.8%) in grade I(0 TSC-LAM, 6 S-LAM), 27 cases (44.3%) in grade II(5 TSC-LAM, 22 S-LAM), and 28 cases (45.9%) in grade III (5 TSC-LAM, 23 S-LAM). Abdominal CT scans found extrapulmonary involvements in LAM patients including renal angiomyolipoma in 29 (47.5%) patients (8 TSC-LAM, 21 S-LAM), pelvic uterine lymphangiomyomas in 13 (21.3%) patients (0 TSC-LAM, 13 S-LAM), liver hamartoma in 11 (18%) patients (3 TSC-LAM, 8 S-LAM), and retroperitoneal lymphangiomyomas in 9 (14.8%) patients (1 TSC-LAM, 8 S-LAM). It is interesting to notice that the percentage (80.0%) with renal angiomyolipomas in TSC-LAM is significantly different from that (41.2%) in S-LAM (*P<0*.*05*).

The pathological findings showed characteristic features of LAM in all the analyzed tissues, such as cystic lesions in pulmonary tissues, multiple immature smooth muscle cells and perivascular epithelial cells in pulmonary and other tissues. Multiple immunohistochemical staining was performed showing positive SMA and HMB45 results in all 61 (100%) cases as well as positive ER (9 TSC-LAM, 38 S-LAM) and PR (8 TSC-LAM, 39 S-LAM) results in 47 (77%) cases respectively.

Serum VEGF-D levels were determined by enzyme-linked immunosorbent assay in all patients in this study at their first visits pursuing diagnosis and treatments. The average serum VEGF-D level were 1,689 pg/ml from TSC-LAM patients and 1,570 pg/ml from S-LAM patients, respectively. Thirty-eight (62.3%) patients had serum VEGF-D levels >800 pg/ml as supporting evidence for the diagnosis of LAM disease in these patients.

### Genetic variants in the *TSC1* and *TSC2* genes in the LAM patients

A combined use of methods (NGS, Sanger sequencing, CMA, and MLPA) identified a total of 86 clinically significant genetic variants of *TSC1/2* in 46 of the 61 LAM patients, with the remaining 15 patients showing negative results, representing an overall positive detection rate of 75.4% (46/61) in which *TSC2* and *TSC1* variants were 88.37% and 11.63% respectively (Fig 1, S4 Table). These results were further organized and analyzed in different ways in following paragraphs.

**Fig 2:**
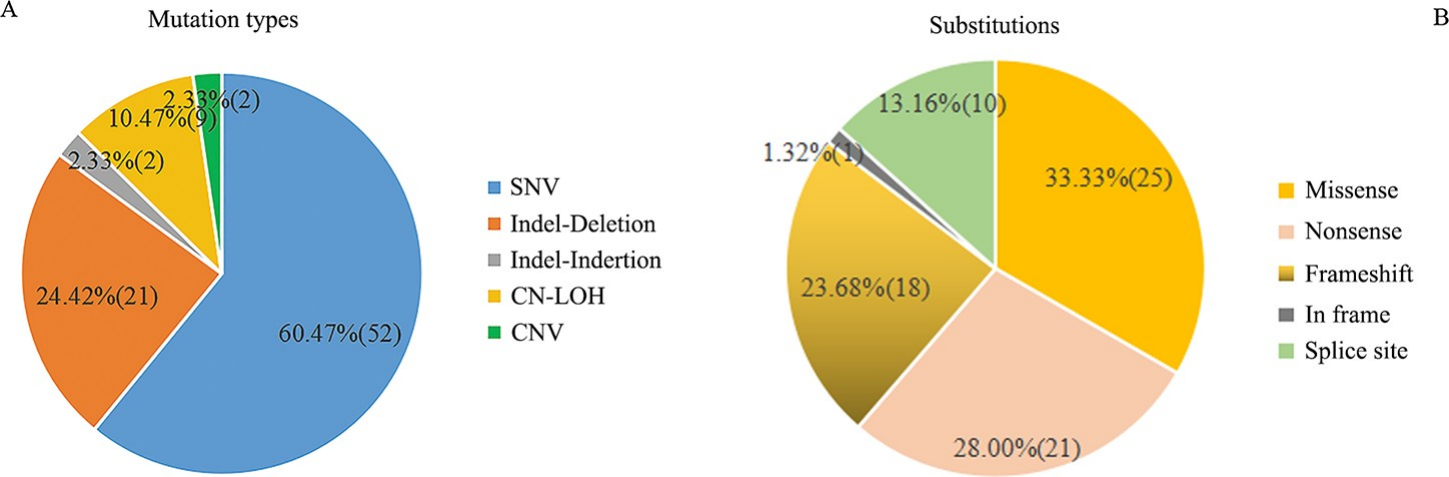
Detection of *TSC1* and *TSC2* variants in the LAM patients. (A)Distribution of the variants identified in this study; (B) Substitutions detected by NGS in *TSC1* and *TSC2*.

**Fig 3:**
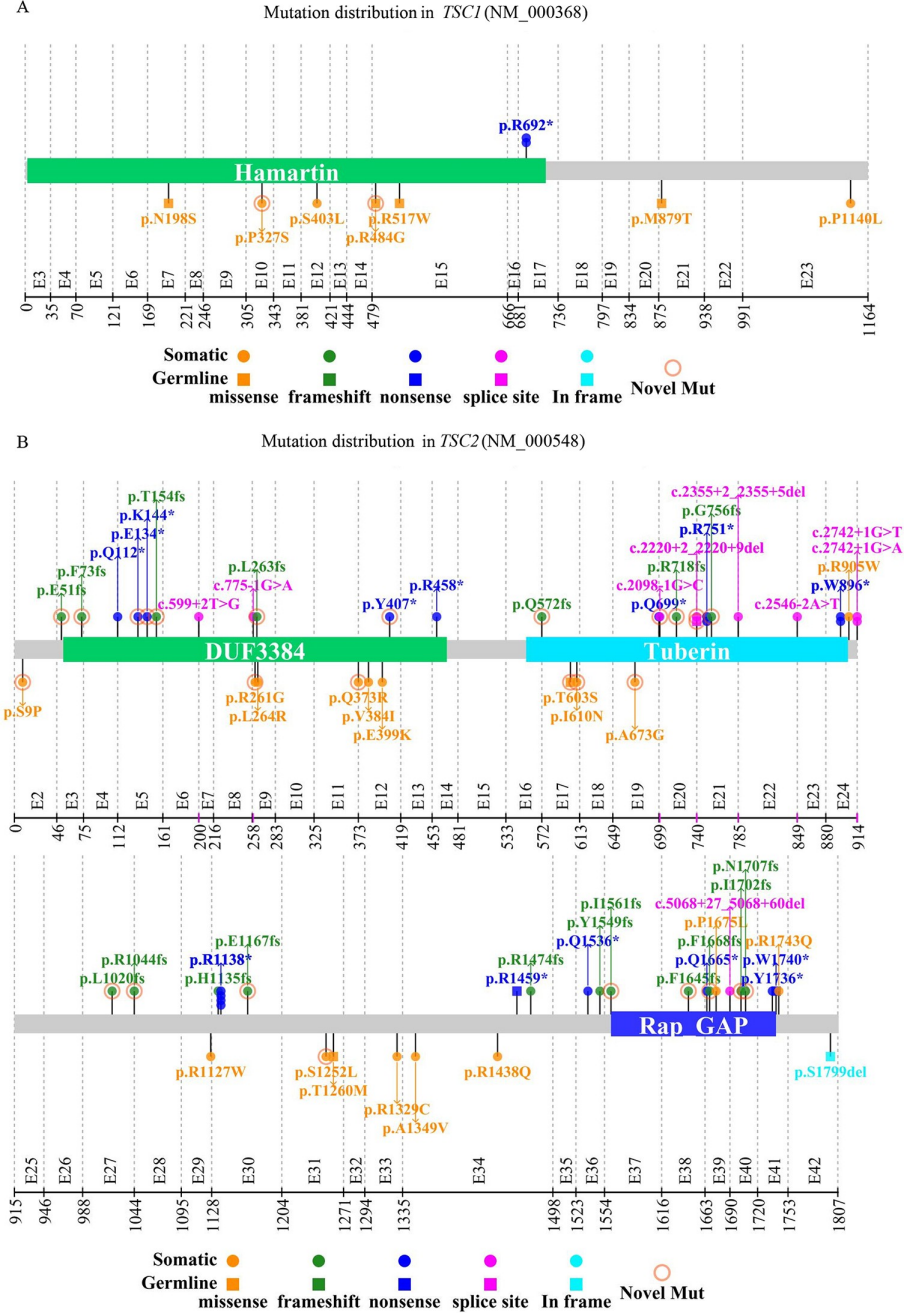
Mutations distribution of *TSC1* and *TSC2*. (A, B) Mutation distribution of *TSC1* and *TSC2* where the variants above the lines were classified as pathogenic (Tier I) or likely pathogenic (Tier II), and the variants below the lines were classified as variants of uncertain significance (Tier III). The variants labeled with a circle are the novel findings.

**Fig 4:**
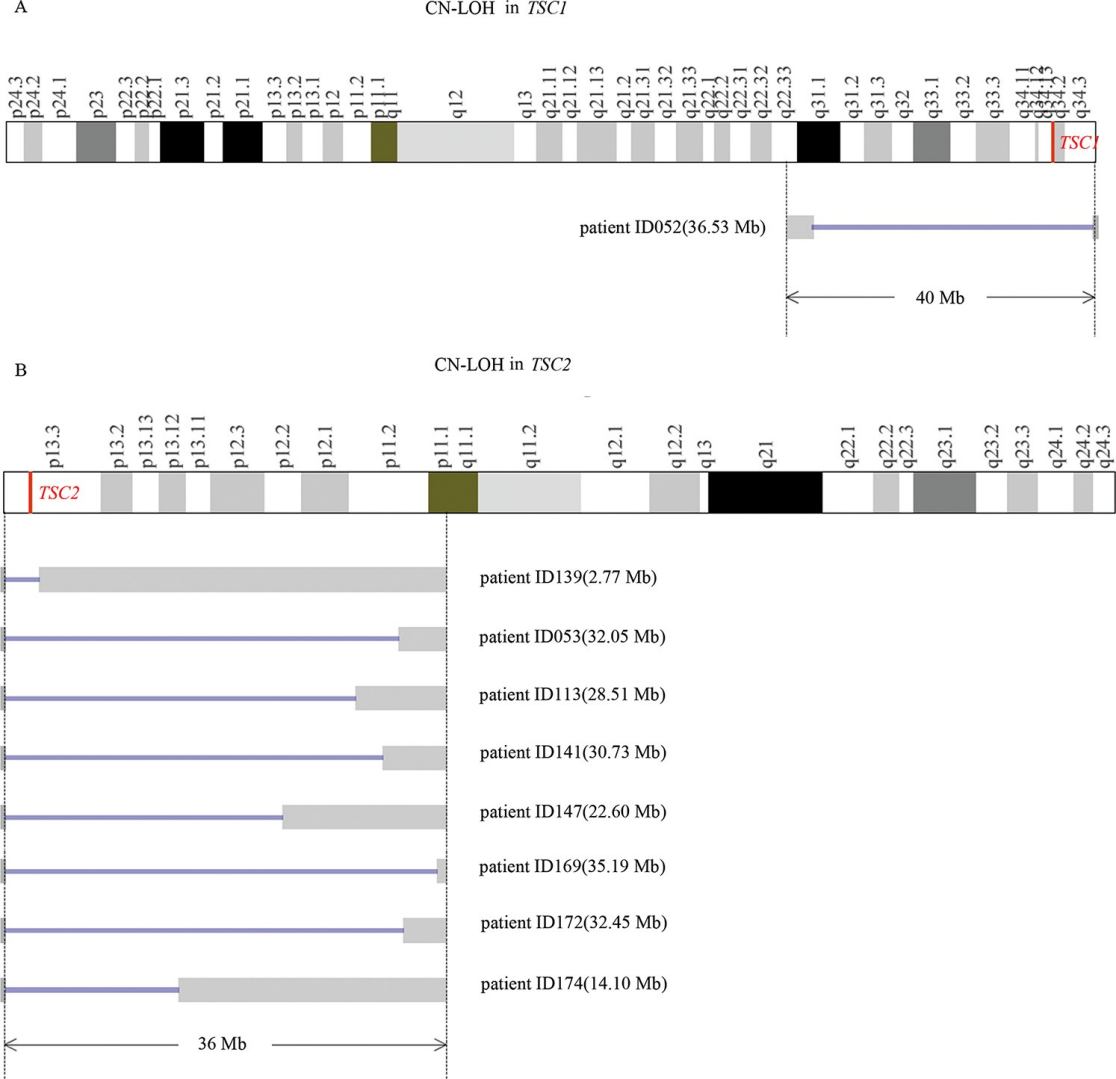
Detection of CN-LOHs in the LAM patients. (A) CN-LOHs involving *TSC1* in LAM patients. (B) CN-LOHs involving *TSC2* in LAM patients. CN-LOH events were always considered somatic, and co-occurred with a somatic mutation identified in the same tissues in our study.

Based on the testing methods used, NGS identified 75 of the 86 variants in 75 biopsies from 44 patients, including 52 single nucleotide variants (SNVs) (9 in *TSC1* and 43 in *TSC2*), 21 small deletions, and 2 small insertions in *TSC2* (Fig 2A and 2B). Among the 75 variants, 45 of them were previously documented in literatures and/or public databases, leaving 30 of the variants as novel findings consisting of 13 SNVs (2 in *TSC1* and 11 in *TSC2*) and 17 indels (0 in *TSC1* and 17 in *TSC2*) (S4 Table, Fig 3A and 3B), there were no significant differences in the distribution of substitution between novel and previously identified in *TSC1/2* (S5 Table) (*P>0*.*05*); MLPA identified a germline duplication of exon 31–42 in *TSC2* in patient ID180; and CMA identified a 2.68 Mb somatic duplication with *TSC2* involved in patient ID160 and 9 CN-LOHs in 9 out of the 24 patients with surplus samples available for testing from the 61 LAM patients (S4 Table, Fig 4A and 4B). Our results showed that all 9 CN-LOHs were present in the LAM patients with single *TSC1/2* mutations but absent in the LAM patients with 2 or more *TSC1/2* mutations or without detectable *TSC1/2* mutations (S5 Table) (*P<0*.*05*). Specifically mentioned here that, in addition to identifying two mutations in *TSC2*, CMA detected complex genomic abnormalities involving multiple chromosomes in patient ID052 with concurrent diagnosis of lung adenocarcinoma (S2 Fig). However, we didn’t find similar chromosomal alterations in other LAM patients in this study.

If a variant was germline-originated, the ACMG guideline was used to determine its clinical significance [18]. If a variant was somatic, the guideline published by Li et al. was used to determine its clinical significance. Meanwhile, Leiden Open Variation and ClinVar Database were highly referenced, CNV and CN-LOH events affecting *TSC1* and *TSC2* were assumed detrimental to gene function. Based on the numbers of *TSC1/2* variants identified in each patient, the 46 patients with 86 significant variants in *TSC1/2* could be categorized into 3 groups: 1) eight patients were found to carry single variants of *TSC1/2* including germline variants in 2 cases (ID140 with a SNV of *TSC2*/p.Arg1459*/Pathogenic and ID180 with a duplication of exon 31–42 of *TSC2*/Likely Pathogenic, however, no obvious symptoms of TSC and no angiomyolipomas or sclerotic bone lesions complications in this patient whom without family history.) and somatic variants in 6 cases (ID030, ID076, ID099, ID060, ID115 and ID162) with lung specimens tested in 7 of the 8 patients except in patient ID115 with a kidney specimen being tested; 2) thirty-six patients were found to carry 2 variants of *TSC1/2* including 1 patient (ID148) with a germline mutation (*TSC1*/p.Met879Thr/VUS) plus a somatic mutation (*TSC2*/p.Arg1438Gln/Tier III), 1 patient (ID131) with a germline mutation (*TSC2*/c.2220+2_2220+9del/Likely Pathogenic) plus a somatic mutation (*TSC2*/p.Ser1252Leu/Tier III), 1 patient (ID052) with a germline mutation (*TSC2*/p.Thr603Ser/VUS) plus a CN-LOH (*TSC1*), 1 patient (ID187) with 2 VUS germline mutations occurred in the same allele of *TSC1* (p.Arg517Trp/p.Arg484Gly), 5 patients (ID015-uterus, ID023-lung1, ID058-lung1&2, ID137-lung, ID153-lung) carrying 2 somatic mutations with one of them in *TSC1* and the other in *TSC2* (Of the 5 patients carrying both *TSC1/2*, all of the 5 *TSC2* variants were classified as Tier I or Tier II while all of the *TSC1* variants were classified as Tier III), 8 patients with a somatic (*TSC2/*Tier I or Tier II) plus a CN-LOH (*TSC2/*Tier I), and 19 patients carrying 2 somatic mutations in the same gene of *TSC2*; 3) two patients were found to carry 3 mutations including patient ID152 and ID154 carrying a germline (*TSC2*) plus 2 somatic mutations (*TSC2*) respectively, the germline mutations in patient ID152 (*TSC2*/p.Ser1799del) and ID154 (*TSC2*/p.Thr1260Met) were classified as VUS while the two *TSC2* somatic mutations were considered to be Tier I or Tier II variants. Detailed information about these genetic variants was listed in S4 Table.

Based on the genetic findings in *TSC1/2*, correlations between genotypes and clinical diagnosis (TSC-LAM or S-LAM) for the 61 patients were analyzed. Eleven of the 86 *TSC1/2* variants were determined to be germline mutations (4 in *TSC1* and 7 in *TSC2)* while the remaining 75 variants occurred somatically. The 11 germline mutations were present in 10 patients including 1 patient (ID187) carrying 2 mutations in one allele. Eight and 2 of the 10 patients were clinically diagnosed as TSC-LAM and S-LAM respectively. A total of 9 germline mutations were detected in 8 of the 10 TSC-LAM patients, including 2 pathogenic mutations in patients ID140 (*TSC2*/p.Arg1459*) and ID130 (*TSC2*/p.Trp1740*), 1 Likely Pathogenic mutations in ID131 (*TSC2*/c.2220+2_2220+9del), and 6 VUS: ID052 (*TSC2*/p.Thr603Ser), ID058 (*TSC1*/p.Asn198Ser), ID148 (*TSC1*/p.Met879Thr), ID152 (*TSC1*/p.Ser1799del) and ID187 (*TSC1*/p.Arg517Trp, *TSC1*/p.Arg484Gly). In addition, in 1 (ID140) of the 10 patients with clinical diagnosis of TSC-LAM, only a germline mutation in *TSC2* (first hit) was found but failed to find the second somatic mutation (second hit) in her genome. Conversely, no germline variant was detectable in the 2 patients with clinical diagnosis of TSC-LAM although somatic mutations were found in both of them, patient ID076 carrying a somatic nonsense mutation in *TSC1* and patient ID183 carrying 2 somatic mutations in *TSC2* (a deletion of exon 34 and an insertion of exon 41 in *TSC2* from two separate kidney biopsies and both of them causing frameshift mutations). Taken together, all the 10 patients (100%) with clinical diagnosis of TSC-LAM were found to carry *TSC1/2* mutations while only 36 of the 51 (70.6%) patients with clinical diagnosis of S-LAM were found to carry *TSC1/2* mutations, with a statistically significant difference between the positive detection rates in these two subgroups (*P<0*.*05*).

### Genetic variants in the non-*TSC1/2* genes in LAM patients

**Fig 5:**
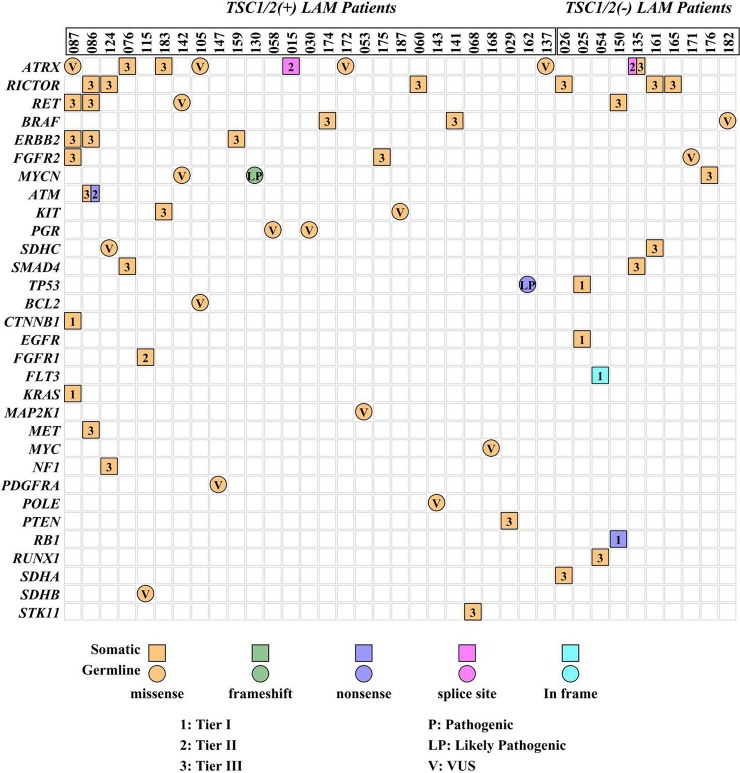
Distributions of cases carrying non-*TSC1/2* variants identified in this study.

In addition to the 86 significant genetic variants identified in *TSC1* and *TSC2*, 61 variants in 31 non-*TSC1/2* genes related to tumorigenesis were found in the 37 LAM patients, including 1 frame-shift deletion (ID130), 1 in-frame insertion (ID054), 3 nonsense mutations (ID086, ID150, and ID162), 2 splicing mutations (ID015 and ID135), and 54 missense mutations (S6 Table, Fig 5). According to the published guidelines and related database LOVD2.0 [16–20], the 61 non-*TSC1/2* variants were further categorized into 3 groups based on their clinical significance, including 6 pathogenic (Tier I), 6 likely pathogenic (Tier II), and 49 VUS (Tier III) (S6 Table). Of the 12 variants classified as pathogenic or likely pathogenic, their allele frequencies were not found in the public population databases and were ≤0.01% present in our internal database deriving from more than 30,000 Chinese individuals.

Based on their cell origins, the 61 non-*TSC1/2* variants were grouped into 20 germline and 41 somatic mutations. The 20 non-*TSC1/2* germline variants were mutated in 15 genes in 18 LAM patients, with 3 genes mutated in at least 2 patients, including *ATRX* in 4, *MYCN* in 2, and *PGR* in 2. Two patients carried 2 non-*TSC1/2* germline variants, patient ID105 with mutations in *ATRX* and *BCL2* and patient ID142 with mutations in *MYCN* and *RET* (S7 Table). The allele frequency of the 20 non-*TSC1/2* germline variants in this cohort are significantly higher than their frequencies of ≤0.01% present in our internal database deriving from more than 30,000 Chinese individuals and publicly available databases (*P<0*.*05*), The variants found in patient ID162 (*TP53*/p.Gln52*) and ID130 (*MYCN*/p.Asp264Glufs*53) were considered Likely Pathogenic and the others were considered VUS.

Forty-one non-*TSC1/2* somatic variants in 24 genes were found in 22 LAM patients and the mutations per patient ranged from 2 to 6. The allele frequencies of the somatic variants were either not found or ≤0.2% present in the public population databases (1000g, ExAC, gnomAD, etc.). Their clinical significances were classified according to published guidelines as described above. We found 6 Tier I mutations in 4 patients (ID025/kidney/*EGFR*/p.Leu858Arg and kidney/*TP53*/p.Arg175His, ID054/lung/*FLT3*/p.Pro606_Phe612dup, ID087/lung/*CTNNB1*/p.Ser45Phe and *KRAS*/p.Gly12Val, ID150/lung/*RB1*/p.Arg556*). Three of the 4 patients were found without significant variants in *TSC1/2*. Besides, 4 Tier II mutations were found in 4 patients (ID015/uterus/*ATRX*/c.4809+1G>A, ID086/lung/*ATM*/p.Gln2881*, ID115/kidney/*FGFR1*/p.Asn546Lys, ID135/lung/*ATRX*/c.6218-1G>A), and all of the other 31 mutations were considered to be Tier III variants (S8 Table). Of them, eight non-*TSC1/2* genes were mutated in at least 2 LAM patients, *RICTOR* in 6, *ATRX* in 5, *ERBB2* in 3, *RET* in 3, *ATM* in 2, *BRAF* in 2, *FGFR2* in 2, and *SMAD4* in 2. All the 6 *RICTOR* variants observed in this study were somatic while none of them was germline-generated (*P<0*.*05*). One (ID135) of the 15 LAM patients without detectible *TSC1/2* variants carries two somatic variants in *ATRX*, and currently it is unknown whether they are on the same allele or on different alleles. Meanwhile, a recurrent variant *ATRX*/p.Arg907Gln were present in both patients ID087 and ID183 respectively.

## Discussion

According to the Knudson’s “two-hit” theory [11,12], TSC-LAM is supposed to carry one germline mutation (first hit) and an additional acquired mutation (second hit) while S-LAM is supposed to carry two acquired mutations in *TSC1/2* [13,14]. In the current study, although only 24.6% (15/61) of the LAM patients were absent of pathogenic or likely pathogenic *TSC1/2* variants in their genomes, 47.5% (29/61) of them was considered as lack of detectable biallelic inactivation of *TSC1/2*, including 8 patients with single pathogenic or likely pathogenic *TSC1/2* variants, 6 patients with one variant in *TSC1* and the other in *TSC2*, and 15 patients without any detectable clinically relevant variants in *TSC1/2*. Based on the results from ours and others, we think the reasons leading to lack of detectable *TSC1/2* mutations in some LAM patients could be categorized into two groups: technical reasons in which the methods used failed to identify the *TSC1/2* mutations present in LAM patients and biological reasons in which mutations in some non-*TSC1/2* genes, independently or synergizing with *TSC1/2*, might have contributed to tumorigenesis of S-LAM.

### Technical improvements required to maximize the detection rate for *TSC1/2* mutations

Currently, no single test method could identify all genetic alterations. For example, of the 86 variants involving *TSC1/2* identified in this study, the target NGS identified 75 SNVs and indels, CMA identified 9 CN-LOHs and a duplication, and MLPA identified a duplication, emphasizing the significance of combining different genetic methods for achieving maximal detection rate of *TSC1/2* mutations in LAM patients. It is interesting to notice that all 9 CN-LOHs were present in the LAM patients with single *TSC1/2* mutations, but absent in the LAM patients with 2 or more *TSC1/2* mutations or without detectable *TSC1/2* mutations (S5 Table) (*P<0*.*05*), suggesting that mitotic formation of somatic CN-LOHs involving *TSC1/2* is a common mechanism leading to biallelic inactivation of *TSC1/2* as the underlying cause of LAM.

The VAF of a mosaic mutation in *TSC1/2* might be present, but lower than the cutoff value (1%) of sequencing reads set in our study, requiring more sensitive sequencing method to unveil them. Alternatively, instead of improving the sensitivity in a testing method, Laser Capture Microdissection (LCM) techniques, an approach to enriching the LAM cells in the biopsy tissues could reach relatively higher detection rates by picking up some mutants of low sequencing reads [25,26];

Mutations located within intronic regions of *TSC1/2* were not covered by our target NGS and if the mutations did have occurred and might have been missed by our methods. Tyburczy et al. reported that intronic mutations in *TSC1/2* might explain some of the previously unidentified *TSC1/2* mutations in TSC patients [27]. Currently, we don’t have enough DNA to further test whether some mutations might be present in the non-coding regions of *TSC1/2* in the patients. Thus, in the future, it is ideal to sequence whole genes covering both exonic and intronic regions of *TSC1* and *TSC*2 [27,28];

### Further evidence required to elucidate the associations between mutations in some non-*TSC1/2* genes and tumorigenesis of LAM

In the current study, all the 10 patients with clinical diagnosis of TSC-LAM were found to carry *TSC1/2* mutations and 8 of them carried biallelic inactivation of *TSC1/2*, suggesting that it is less like that a non-*TSC1/2* tumor suppressor gene might have participated in the initiation of TSC or TSC-LAM, consistent with the opinion from Lam HC et al. that “the possibility of a third germline TSC-causing gene is unlikely” in patients with TSC [26,27,29]. Lam HC et al.’s opinion was based on multiple studies that the majority of this kind of patients have either a genetically mosaic form or an intronic mutation of *TSC1/2* that could not have been detected using conventional screening methods. In contrast to the fact that all of the 10 patients with clinical diagnosis of TSC-LAM were found to carry *TSC1/2* mutations, only 36 of the 51 (70.6%) patients with clinical diagnosis of S-LAM were found to carry *TSC1/2* mutations (*P<0*.*05*), indicating some non-*TSC1/2* mutations might have participated in the initiation of S-LAM. Interestingly, 6 somatic mutations in the *RICTOR* gene were identified in 6 S-LAM patients separately in this study and somatic *RICTOR* mutations related to stomach and intestine cancers were documented in the COSMIC database. The *RICTOR* (rapamycin-insensitive companion of mTOR) gene encodes a core component of the mTOR complex-2 (mTORC2) and recent findings suggested that high mTORC2 activity may play a role in the pathogenesis of LAM [10,30]. Apparently, the functional significance of these *RICTOR* mutations need further investigation. Another interesting finding in the current study is that 9 *ATRX* variants (4 germline and 5 somatic variants) were identified, representing the most common non-*TSC1/2* variants in the 61 LAM patients, including one (ID135) of the 15 LAM patients without detectible *TSC1/2* variants but with two somatic variants of *ATRX*, and a recurrent variant (*ATRX*/p.Arg907Gln) present in both patients ID087 and ID183 respectively. *ATRX* is a tumor suppressor gene located on chromosome X and biallelic inactivation of this gene through mutations or chromosome X inactivation is associated with tumorigenesis in a variety of tumors. *ATRX* encodes a protein required for suppression of telomeric repeat-containing RNA expression [31]. The underlying mechanism linking *ATRX* mutations and initiation and progression of LAM diseases is unknown currently.

### Genetic and functional studies required to characterize the correlations between *TSC1/2* variants and phenotypes of LAM

Interaction of trans-heterozygous *TSC1/2* mutations between a mutated *TSC1* and a mutated *TSC2* might have contributed to tumorigenesis in the 6 patients with one variant in *TSC1* and the other in *TSC2* in this study. Similar cases were previously reported in the tumor tissues of patients with TSC [32]. Uhlmann EJ reported that *Tsc1 + /-*; *Tsc2 +/-* compound heterozygous mice show potential epistatic interaction between monoallelic mutation of the two genes [33,34];

Among the 36 patients with clinical diagnosis of S-LAM, patient ID180 carries a novel germline duplication of exon 31–42 in *TSC2* without clinical detectable symptom of TSC. Similarly, Patient ID154 with a diagnosis of S-LAM carries 2 *TSC2* variants, a germline variant identified in the blood and 2 somatic variants in her lung tissue. Possible explanation is that the symptoms of TSC in these patients were too mild to be observed routinely. Similar results were reported before, for example, Sato et al identified a novel *TSC1* germline mutation in 1 (4.5%) of the 22 S-LAM patients [35]. These results lead us to speculate that some of the patients with clinical diagnosis of S-LAM might carry atypical germline mutation of *TSC1/2*, suggesting that genetic testing for *TSC1/2* is critical to the clarification of a clinical diagnosis of LAM disease.

In patient ID187 with a diagnosis of TSC-LAM, NGS identified 2 germline mutations in the exon 15 of *TSC1 in cis (*on the same allele) and both were categorized as Tier III variants (VUS). Theoretically, it is less likely to have two causing mutations occurring on the same allele and both them are driver mutations causing initiation of tumorigenesis. Thus, it is reasonable to speculate that at least one of the two germline mutations may not have effect on the initiation of TSC-LAM in this patient. This speculation is probably applicable to some of the other VUSs present in *TSC1/2* identified in this study. Thus, further studies are needed to demonstrate the biological functions of the VUSs and their association to the formation of LAM.

### Limitations of the current study

We acknowledge that there are some limitations in this study, including: 1) published results showed that a third of the *TSC1/2* mutations in patients with TSC were inherited and two thirds occurred *de nov*o [36–38]. Lack of pedigree information in some patients carrying germline mutations of *TSC1/2* restrained us to determine whether the germline mutations might have inherited from one of the parents or occurred *de novo*. 2) Limited quantity of DNA samples in some patients restrained us to implement further genetic testing; 3) Although some identified novel variants of *TSC1/2* were considered to be the likely causes of S-LAM in these patients, further investigations about these variants are required to determine their abnormal functions and pathogenicity underlying the formation of LAM; and 4) the non*-TSC1/2* somatic mutations identified in the LAM patients need to be further investigated about their association to the tumorigenesis of LAM.

## Conclusion

A wide spectrum of genetic alterations in *TSC1/2* identified in the cohort supports clinical applications of a combined use of different techniques in order to achieve maximal molecular detection rate for LAM patients. Thirty novel variants in *TSC1/2* identified contributes to the *TSC1/2* data resources underlying the cause of LAM, valuable for the diagnosis and subsequent treatment of the disease. Identification of a long list of non-*TSC1/2* variants improve our understanding about the genetic abnormalities relevant to tumorigenesis of LAM, especially with our suggestion that alternative mechanisms might have contributed to the tumorigenesis and development of LAM.

## Supporting information

S1 Fig Chest CT scans of patient ID052. (DOC)

S2 Fig Complex genomic abnormalities involving multiple chromosomes in patient ID052 by CMA. (DOC)

S1 File The details of supplementary figures. (DOC)

S2 File The details of supplementary tables. (XLSX)

S1 Table Clinical parameters of the 61 LAM patients. (XLSX)

S2 Table Baseline demographic and clinical characteristics of the patients. (XLSX)

S3 Table Custom gene panel designed for target sequencing. (XLSX)

S4 Table Genetic variants in *TSC1/2* in the LAM patients. (XLSX)

S5 Table Relevant statistical details in this study. (XLSX)

S6 Table Total genetic variants in non-*TSC1/2* identified in the LAM patients. (XLSX)

S7 Table Germline variants in non-*TSC1/2* identified in the LAM patients. (XLSX)

S8 Table Somatic variants in non-*TSC1/2* identified in the LAM patients. (XLSX)
