# Correction: Mutation spectrums of *TSC1* and *TSC2* in Chinese women with lymphangioleiomyomatosis (LAM)

**DOI:** 10.1371/journal.pone.0228066

**Published:** 2020-01-15

**Authors:** Jie Liu, Weiwei Zhao, Xiaohua Ou, Zhen Zhao, Changming Hu, Mingming Sun, Feifei Liu, Junhao Deng, Weili Gu, Jiaying An, Qingling Zhang, Xiaoxian Zhang, Jiaxing Xie, Shiyue Li, Rongchang Chen, Shihui Yu, Nanshan Zhong

The following information is missing from the Funding statement: This study was supported by grants from The National Key Research and Development Program of China (2016YFC0901502) to JL, Medical Science and Technology Research Grant of Guangdong Province (A2015018) to JL, Science and Technology Program of Guangzhou, China (201604046001) to WZ, Project of Academician Workstation at KingMed Dianostic (2017B090904030) to WZ, and Program for Entrepreneurial and Innovative Leading Talents of Guangzhou, China (CXLJTD-201603) to SY.
