# Supplementary figures and images for: Mutation spectrums of TSC1 and TSC2 in Chinese women with lymphangioleiomyomatosis (LAM)
Source: PLoS One. 2019 Dec 19;14(12):e0226400. doi: 10.1371/journal.pone.0226400 (PMC6922431; doi:10.1371/journal.pone.0226400)

**S1 Fig.**

| **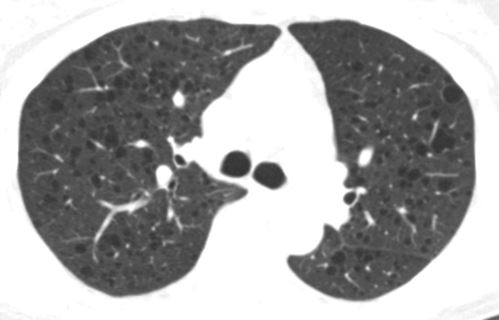** | **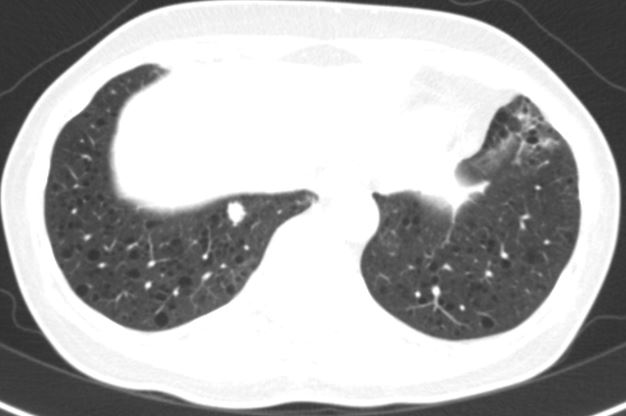** |
| --- | --- |

Supplement: S1 Fig — (DOC) [file pone.0226400.s001.doc]
