# Supplementary material for: Mutation spectrums of TSC1 and TSC2 in Chinese women with lymphangioleiomyomatosis (LAM)
Source: PLoS One. 2019 Dec 19;14(12):e0226400. doi: 10.1371/journal.pone.0226400 (PMC6922431; doi:10.1371/journal.pone.0226400)
Supplement: S2 Fig — (DOC) [file pone.0226400.s002.doc]

**S2 Fig.**

| 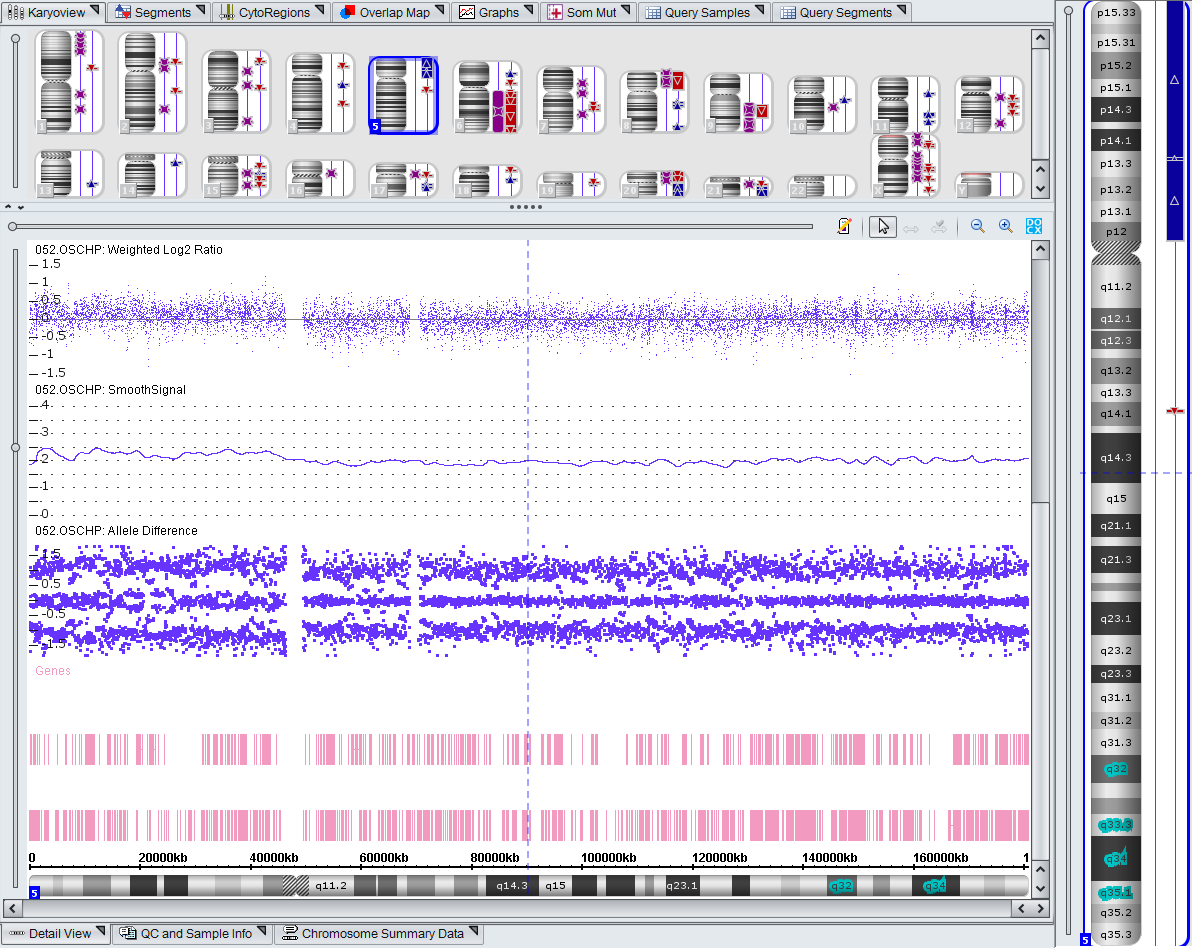 | 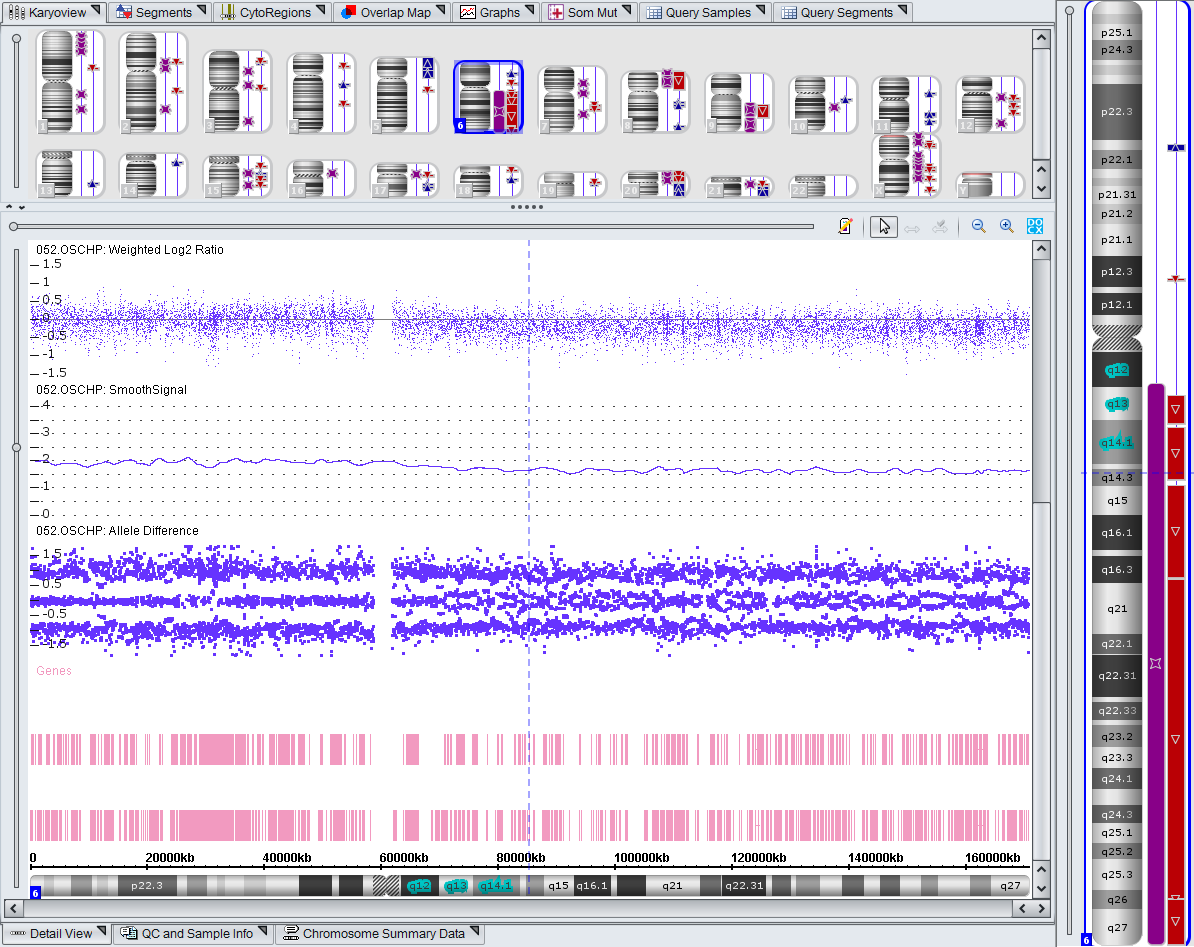 |
| --- | --- |
| 1. arr[GRCh37] 5p15.33p12(38138_46062889)x3[0.3] 46.02Mb | B. arr[GRCh37] 6q13q27(71623252_170913051)x1[0.3] 99.29Mb |
| 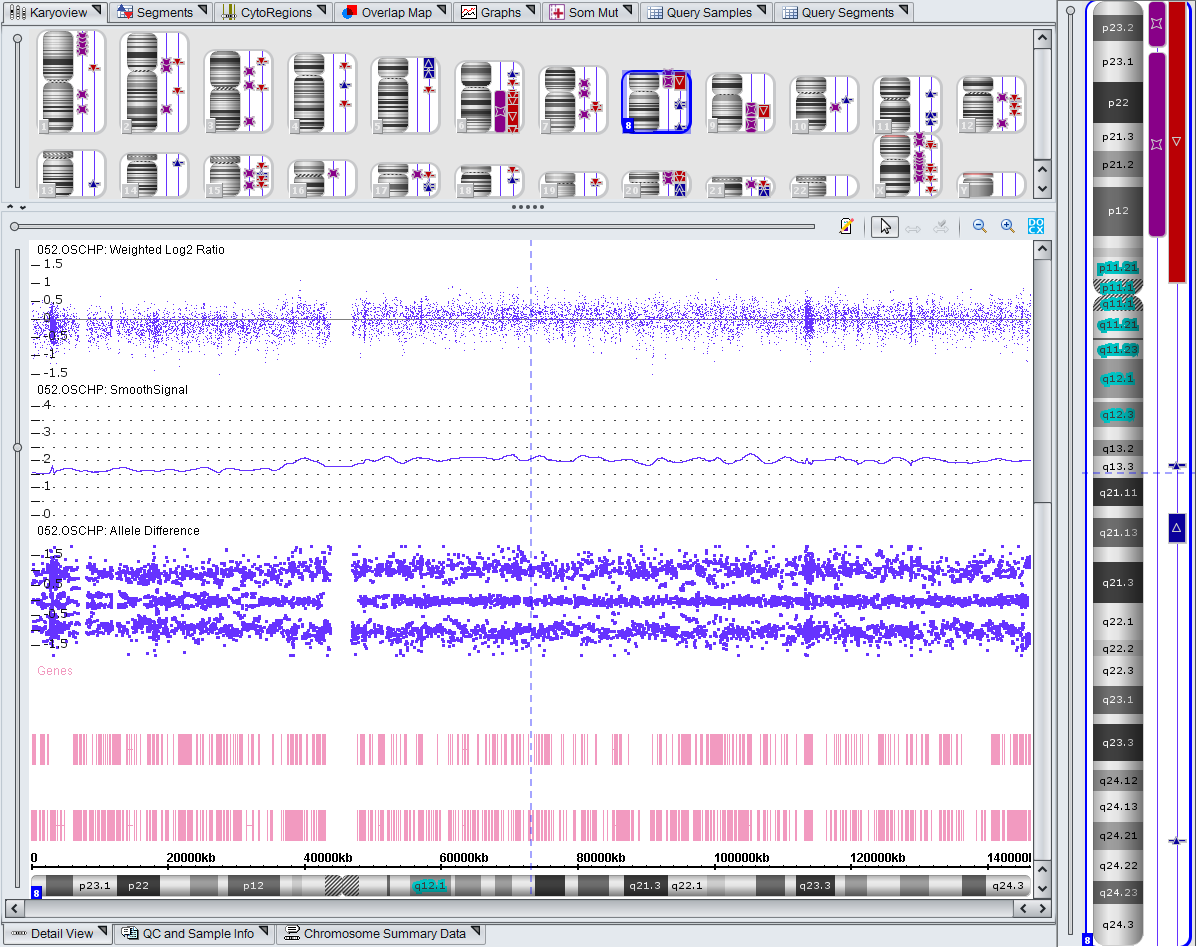 | 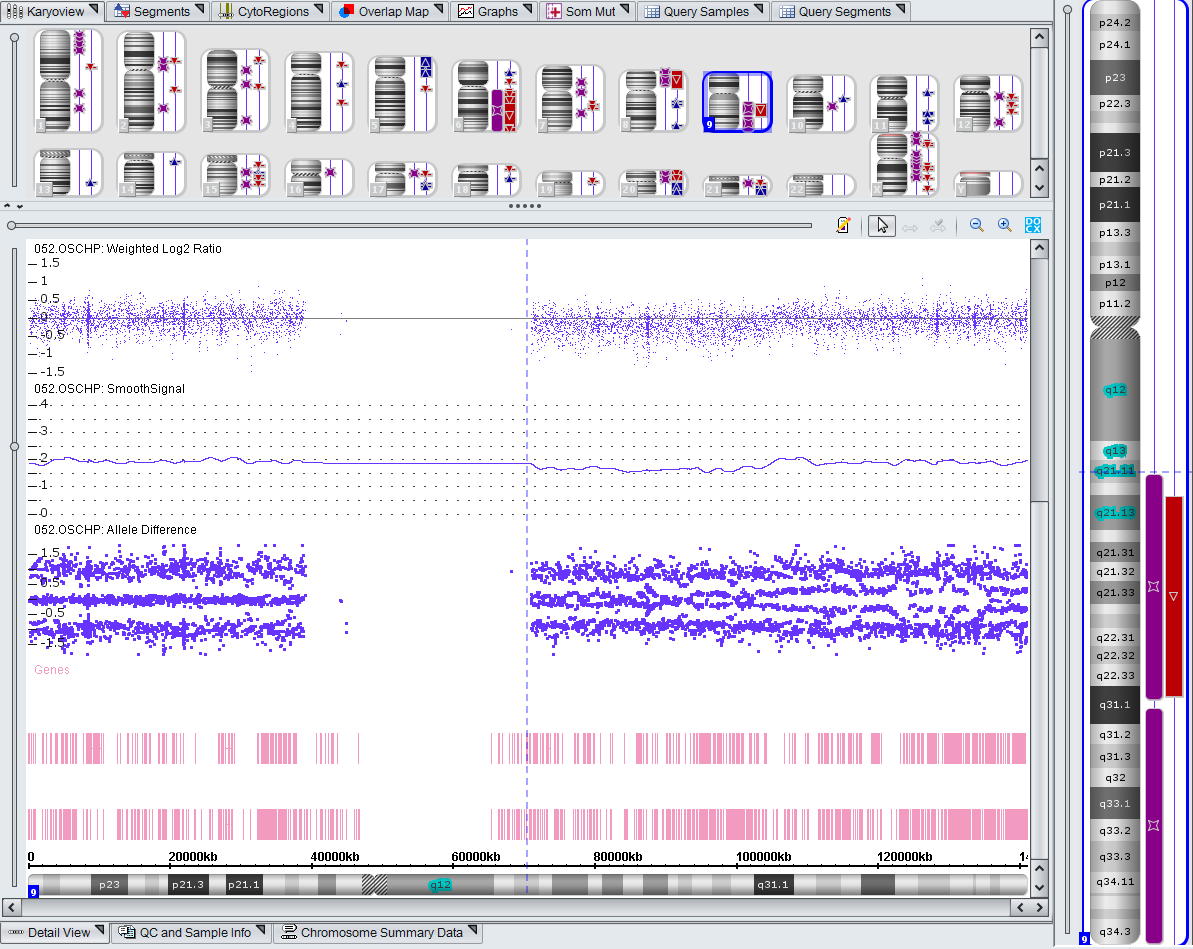 |
| C. arr[GRCh37] 8p23.3p12(172416_36446409)x1[0.3] 36.27Mb | D. arr[GRCh37] 9q21.11q31.1(70984371_104522836)x1[0.3] 33.54Mb  arr[GRCh37] 9q31.1q34.3(104522836_141054761)x2 mos hmz 36.53Mb |
| 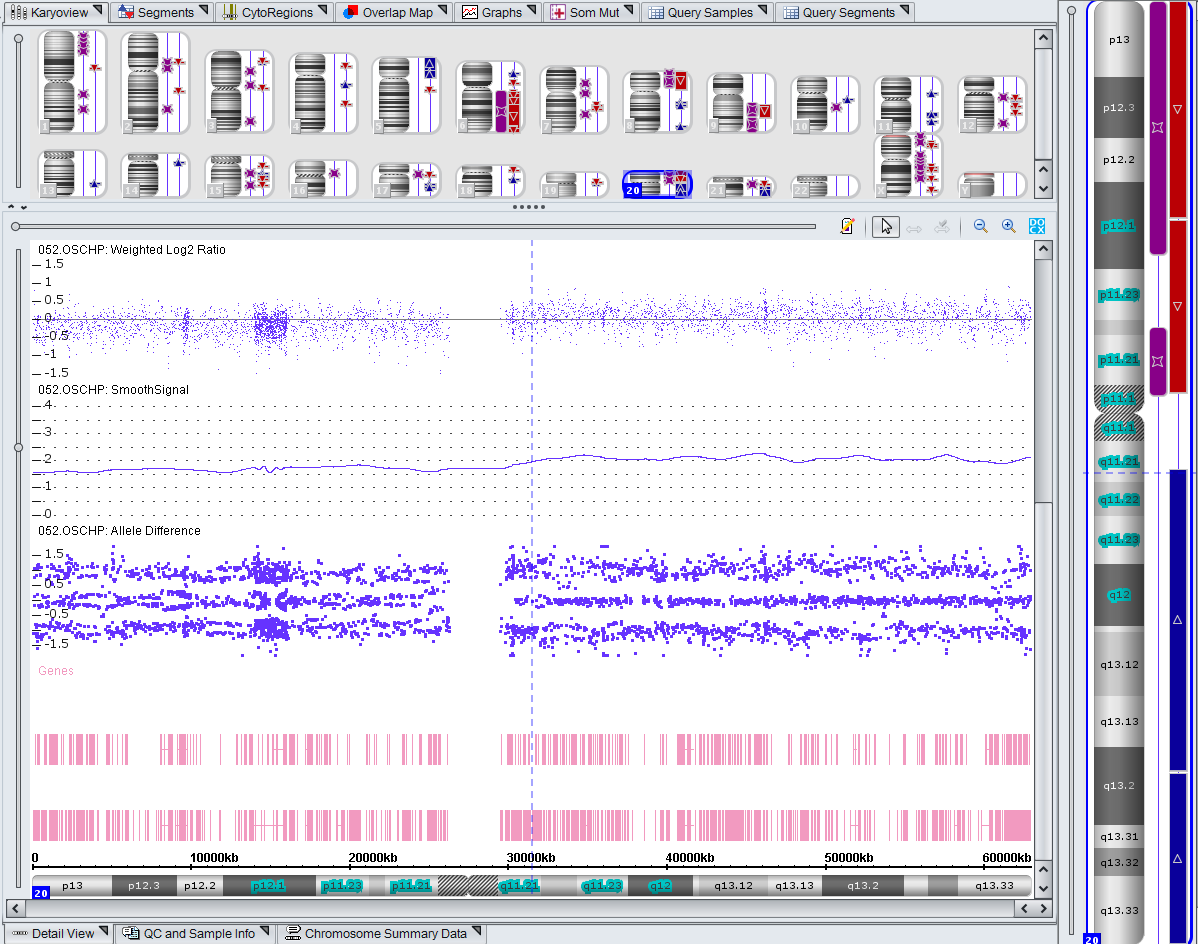 | 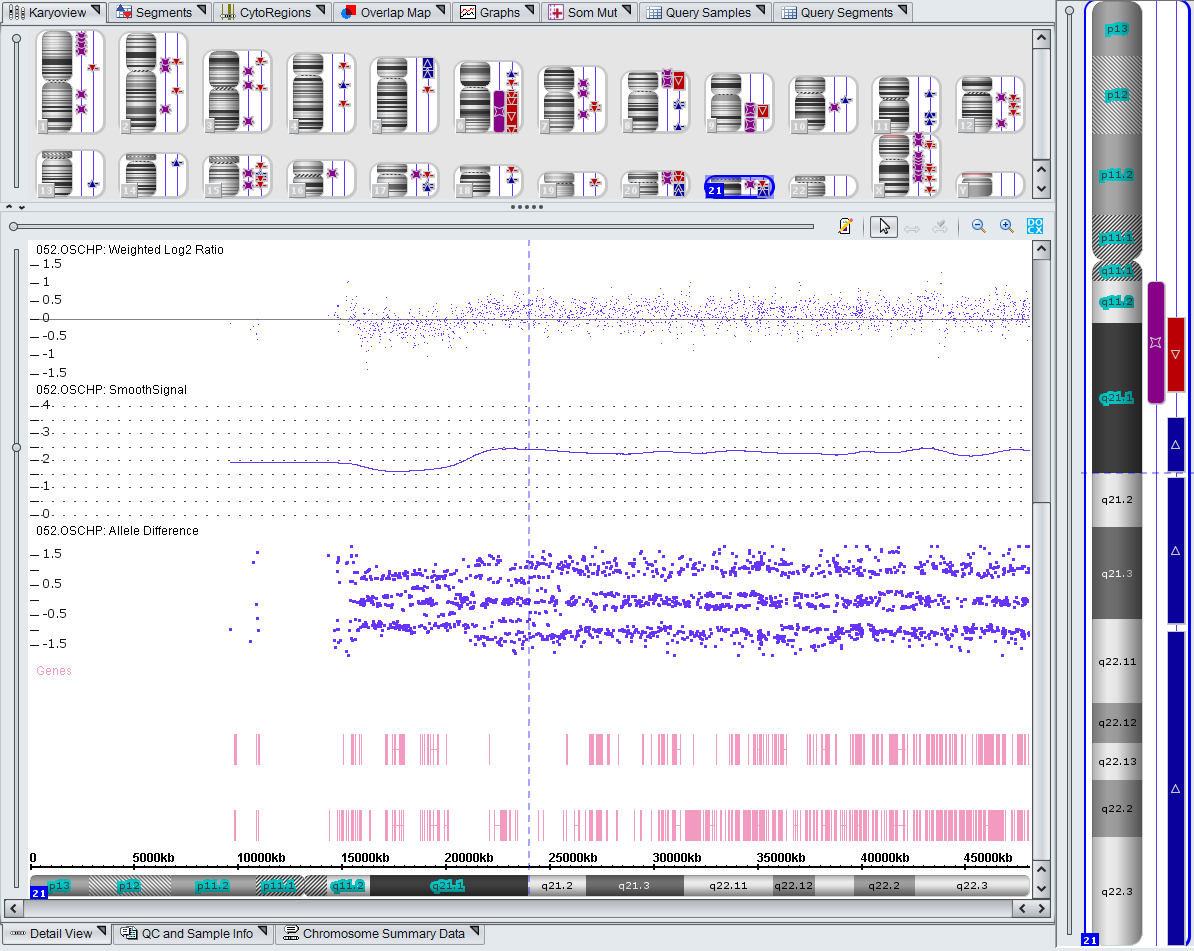 |
| 1. arr[GRCh37] 20p13p11.1(69093_26151507)x1[0.3] 26.08Mb   arr[GRCh37] 20q11.21q13.33(31308683_62912463)x3[0.3] 31.60Mb | 1. arr[GRCh37] 21q11.2q21.1(16157706_21256578)x1[0.3] 5.10Mb   arr[GRCh37] 21q21.1q22.3(21256578_48097610)x3[0.6] 26.84Mb |
